# Accelerated Wound Border Closure Using a Microemulsion Containing Non-Inhibitory Recombinant α1-Antitrypsin

**DOI:** 10.3390/ijms23137364

**Published:** 2022-07-01

**Authors:** Alon Gimmon, Lior Sherker, Lena Kojukarov, Melodie Zaknoun, Yotam Lior, Tova Fadel, Ronen Schuster, Eli C. Lewis, Eldad Silberstein

**Affiliations:** 1 Department of Clinical Biochemistry and Pharmacology, Faculty of Health Sciences, Ben-Gurion University of the Negev, Beer-Sheva 8410501, Israel; alongimmon@gmail.com (A.G.); sherker@post.bgu.ac.il (L.S.); lenakoj@gmail.com (L.K.); melodyzaknoun@gmail.com (M.Z.); matoyster@gmail.com (Y.L.); tovafadel@gmail.com (T.F.); ronen.schus@gmail.com (R.S.); lewis@bgu.ac.il (E.C.L.); 2 Department of Plastic and Reconstructive Surgery, Soroka University Medical Center, Beer-Sheva 8410101, Israel

**Keywords:** corticosteroids, infiltration, inflammation, directed mutation, neutrophils, VEGF

## Abstract

Wound healing requires a non-compromising combination of inflammatory and anti-inflammatory processes. Human α1-antitrypsin (hAAT), a circulating glycoprotein that rises during acute-phase responses and during healthy pregnancies, is tissue-protective and tolerance-inducing; although anti-inflammatory, hAAT enhances revascularization. hAAT blocks tissue-degrading enzymes, including neutrophil elastase; it is, therefore, unclear how wound healing might improve under hAAT-rich conditions. Here, wound healing was examined in the presence of recombinant hAAT (hAAT^WT^) and protease-inhibition-lacking hAAT (hAAT^CP^). The impact of both hAAT forms was determined by an epithelial cell gap closure assay, and by excisional skin injuries via a microemulsion optimized for open wounds. Neutrophilic infiltration was examined after 8 h. According to results, both hAAT forms accelerated epithelial gap closure and excisional wound closure, particularly at early time points. Unlike dexamethasone-treated wounds, both resulted in closed borders at the 8-h time point. In untreated and hAAT^CP^-treated wounds, leukocytic infiltrates were widespread, in hAAT^WT^-treated wounds compartmentalized and in dexamethasone-treated wounds, scarce. Both hAAT forms decreased interleukin-1β and increased VEGF gene expression. In conclusion hAAT improves epithelial cell migration and outcomes of in vivo wounds irrespective of protease inhibition. While both forms of hAAT allow neutrophils to infiltrate, only native hAAT created discrete neutrophilic tissue clusters.

## 1. Introduction

The facilitation of an ideal wound healing process from tissue injury to near-full recovery requires well-coordinated inflammatory and anti-inflammatory processes. Initially, pro-inflammatory agents such as IL-1 are released from injured tissues and facilitate further inflammatory responses and immunocyte infiltration. The same inflammatory response is also responsible for the initiation of inflammatory resolution, as well as cellular proliferation and connective tissue repair [1,2].

Neutrophils are the first immunocytes to infiltrate injury sites. They predominate the wound site in the first 4–6 h. Given their ability to perform oxidative bursts and secrete a variety of tissue-destructive enzymes, neutrophils are known to be highly efficient in decontamination, yet may also induce collateral tissue damage (reviewed in [3]). While conditions that result in too few infiltrating neutrophils raise a risk for lethal infection (e.g., neutropenic patients), hyper-infiltration might slow resolution via a self-perpetuating inflammatory process (e.g., chronic obstructive pulmonary disease [4]).

Following the initial inflammatory process, the inflammatory resolution phase is characterized by an altered gene expression profile of local endothelial cells, keratinocytes and fibroblasts, favoring cell proliferation, differentiation and migration [2]. One example of this process can be seen in the hypoxia-induced expression of vascular endothelial growth factor (VEGF), which leads to angiogenic processes. Another example can be seen in the re-epithelialization of the skin and gut mucosa upon injury [5].

Chronic wounds are clinical entities in which tissue injury fails to heal in an orderly manner. These conditions may arise from the failure of inflammatory response initiation (e.g., concomitant chemotherapeutics, non-steroidal anti-inflammatory drugs and corticosteroids) or by chronic inflammatory wound conditions (e.g., indolent infection, underlying ischemic vascular disease or prolonged presence of sutures) [6,7,8].

Human alpha1-antitrypsin (hAAT) is an endogenous circulating 52-kDa serine protease inhibitor, whose concentrations rise 4–6 fold during the acute inflammatory process. hAAT is mostly known in the context of hAAT deficiency, a genetic condition associated with early-onset pulmonary emphysema and necrotizing panniculitis, which is treated by weekly infusions of serum-purified hAAT [9,10]. However, evidence from recent decades has pointed to hAAT also being a potent anti-inflammatory, immunomodulatory, antibacterial and tissue-protective agent [11,12,13]. Furthermore, recent animal studies demonstrate a protective effect for hAAT therapy in the context of multiple types of ischemia–reperfusion injuries [14,15], promoting re-vascularization alongside skin flap training [9].

Interestingly, despite its well-known anti-elastolytic attribute, hAAT does not necessarily require protease inhibition for exerting beneficial outcomes [16,17]. It is postulated that the globular surface of hAAT acts as a highly conserved docking site for several inflammatory agents [18], and that, paradoxically, it is ultimately the binding of a protease that cleaves and inactivates hAAT.

To date, topical administration of hAAT has not been reported in the literature. Based on a robust and long-standing safety record of intravenous hAAT in humans [19,20], application of recombinant hAAT in the context of acute ear infection has been described [21], and inhaled hAAT has been tested in humans [22]. Recently, a recombinant mutated form of hAAT that lacks the capacity to block proteases was found to be as effective as recombinant wildtype hAAT (hAAT^WT^) in exerting anti-inflammatory outcomes [23]. Termed hAAT^CP^ for the replacement of a cysteine residue with a proline residue at position 232, this recombinant protein was found to exert superior anti-inflammatory processes [23]; however, it has yet to be tested in wound healing models. Considering the apparent discrepancy between its anti-inflammatory function and the lack of an ability to inhibit proteases, it is intriguing to learn how hAAT^CP^ functions as a potential wound-healing agent.

In the present study, topical assignment of hAAT^CP^ was assessed. It was compared to both hAAT^WT^, and to dexamethasone (DEX) therapy. Outcomes of epithelial cell migration were examined in vitro, and, focusing on neutrophilic infiltration, the initial hours post-surgical injury were investigated, testing for relative IL-1β and VEGF expression profiles and exploring concomitant treatment with dexamethasone.

## 2. Results

### 2.1. Epithelial Cells Display Enhanced Migratory Capacity under Haat-Rich Conditions, Further Pronounced under A Non-Protease-Inhibitory Formulation

**Figure 1:**
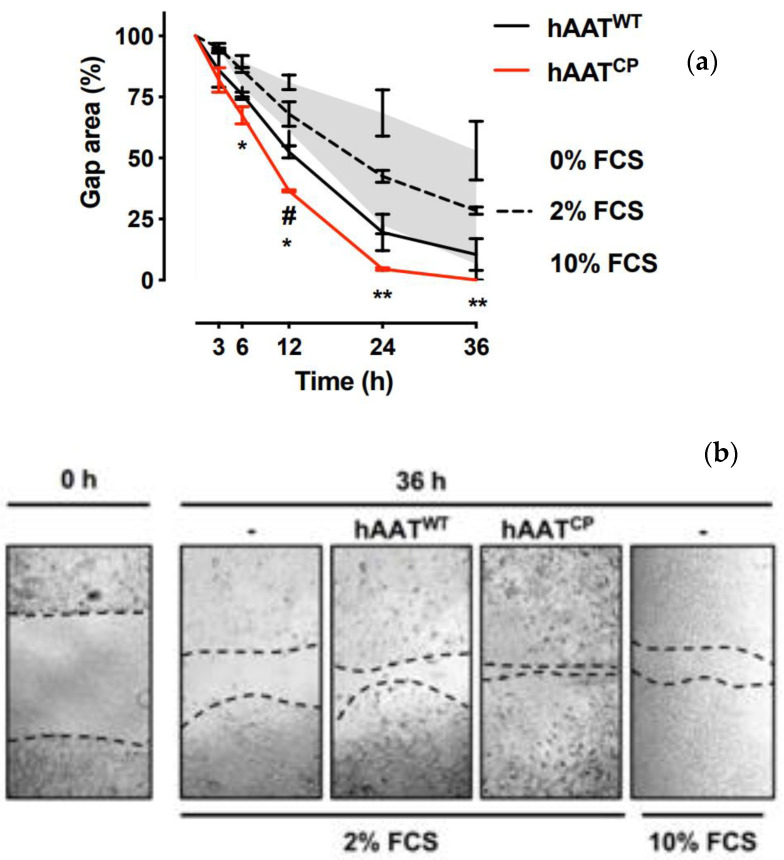
***In vitro epithelial cell gap closure assay***. Monolayers of human epithelial cell line (A549) were disrupted by direct linear scratch, set as time 0. hAAT^WT^ and hAAT^CP^ (each at 200 ng/mL) were added at the time of scratch in 2% FCS. In adjacent wells, cells were cultured in 0% or 10% FCS to signify nutrient-poor or -rich conditions, respectively. Gap area was quantified from culture images acquired at indicated time points, represented as a % of the initial wound area. (**a**) Time course. *Gray*, wound area range between conditions of 0% and 10% FCS per timepoint. *Solid lines*, hAAT-treated wells (in 2% FCS). Mean ± SEM. # *p* < 0.05 between hAAT^WT^ and hAAT^CP^; * *p* < 0.05 and ** *p* < 0.01 between hAAT^CP^ (*red*) and background control 2% FCS (*dashed*). (**b**) Representative photomicrographs (×40 magnification). Wells containing 2% FCS imaged at times 0 and 36 h, added either medium alone (-) or indicated treatments. *Dashed lines*, epithelial borders.

In order to compare the outcomes of treatment with recombinant hAAT^WT^ versus recombinant hAAT^CP^ for epithelial gap repair, confluent A549 cells were uniformly disrupted and treated with 2% FCS in the presence of 200 ng/mL recombinant proteins (Figure 1). As shown, 36 h from scratch, control cells exhibited a gap area that was 28.5% ± 2.12% (mean ± SD) from the initial scratch size. The group treated with hAAT^WT^ depicted accelerated cell migration and a gap area that was 10.5% ± 9.19% from the initial wound size. The group treated with hAAT^CP^ demonstrated complete gap closure (*p* = 0.0028 from 2% FCS). The earliest significant change from gap closure rates at 2% FCS was observed in the hAAT^CP^ group. Examination at 6 h from wounding showed that the gap was reduced from control conditions by 21.52% (*p* = 0.0366). At 12 h from wounding, the gap area of cells treated with hAAT^CP^ was significantly set apart from cells treated with hAAT^WT^ (36.5% ± 0.70% and 52.5% ± 3.53%, respectively, *p* = 0.0245). Notably, the gap area of cells treated with hAAT^CP^ was consistently smaller than that of cells under the ideal condition of 10% FCS.

### 2.2. hAAT Enhances Excisional Skin Wound Closure Irrespective of Protease Inhibition

**Figure 2:**
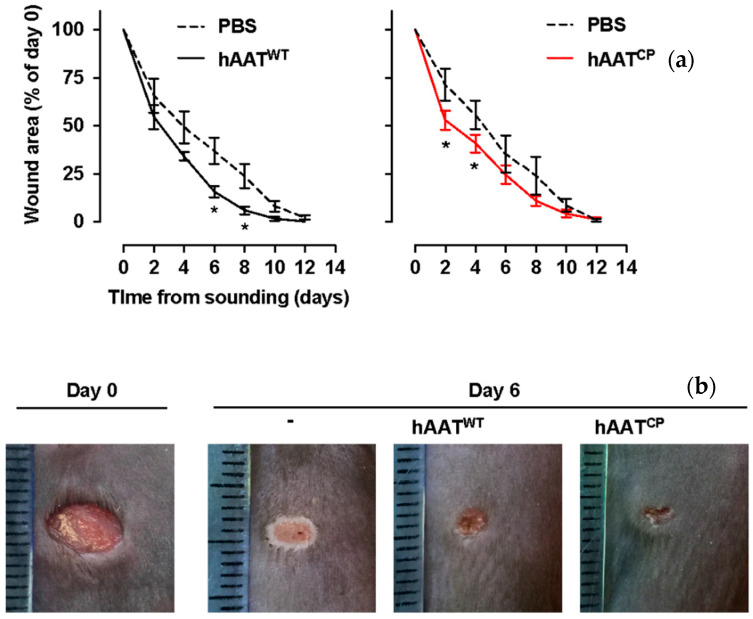
***In vivo excisional skin wound closure***. Animals underwent excisional dorsal wounding (5 mm punch). Treatments included single dermal infiltration of 50 µL PBS alone or containing hAAT^WT^ and hAAT^CP^ (50 µg/wound) at the time of wounding. Wound area was quantified every 2 days, represented as % of the initial wound area. (**a**) Time course. *Dashed line*, control wounds (represented twice to accommodate visual appreciation of treatment outcomes). *Solid lines*, hAAT-treated wounds. Mean ± SEM. * *p* < 0.05 between treatments and control group per time point. (**b**) Representative images of wounds and metric ruler, as acquired on days 0 and 6 of either saline control (-) or treated wounds, as indicated.

Mice underwent excisional dorsal wounding and were treated with infiltration of 50 µL PBS, hAAT^WT^ and hAAT^CP^ into wound edges (Figure 2, n = 6 per group). Wound size was determined serially until day 14. As shown, both hAAT^WT^ (*left*) and hAAT^CP^ (*right*) accelerated wound closure compared to control conditions (represented twice by a *dashed line* in both of the graphs). The earliest time point in which a treatment depicted a significantly accelerated wound closure, compared to the same time point in the control group, was on day 2 in the hAAT^CP^ group (wound area 51% ± 4% compared to 78% ± 5% in controls, *p* = 0.0312). A significant change in the hAAT^WT^ group was observed on day 6 (38% ± 3% compared to 58% ± 6%, *p* = 0.0156). All wounds were fully healed within 14 days of injury.

### 2.3. Neutrophilic Infiltration Profiles Coincide with Subtype of hAAT

**Figure 3:**
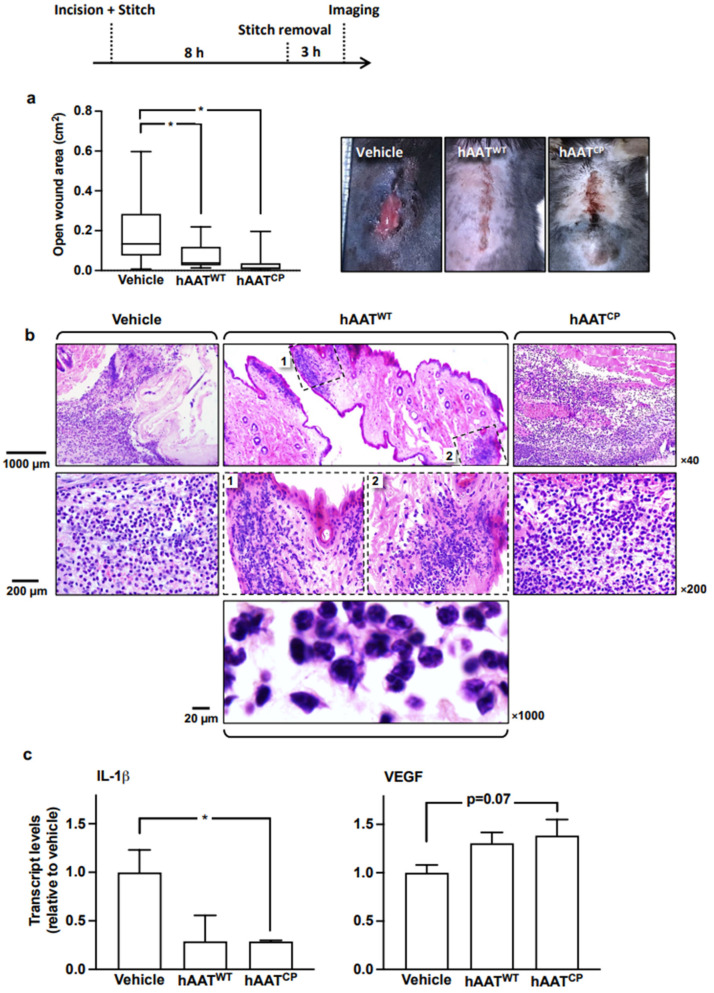
***In vivo wound model: Vehicle compared to hAAT (native and mutated)***. Dorsal incisional wounds were inflicted and then immediately stitched closed (*top, illustration*); 8 h later, stitches were removed and mice were allowed to roam ad libitum for 3 h. Treatments included a single directly added 50 µL PBS alone, or with hAAT^WT^ and hAAT^CP^ (50 µg/wound) at the time of wounding. (**a**) Wound area analysis. *Left*, Median; min–max. * *p* < 0.05. *Right*, representative images of wounds in-frame with a physically placed metric ruler. (**b**) Histology. Representative microscope images (optical magnification and scale indicated); dashed insets are number-matched. (**c**) Gene expression analysis. Mean ± SEM. * *p* < 0.05.

Both epithelial gap closure and in vivo wound closure displayed a response to hAAT treatments that belonged to the early phase of closure, particularly in the case of hAAT^CP^ (Figure 1 and Figure 2). Therefore, the question remains whether the wounds responded in this manner by a more rapid mechanical constriction, or rather by accelerated changes at the cellular level. To examine this, wounds were inflicted and then immediately stitched closed, effectively canceling the mechanical aspect of wound closure (Figure 3 *top*; *illustration*). Eight hours later, stitches were removed, mice were allowed to roam ad libitum, and then the wounds were imaged. Groups included mice treated with the vehicle, hAAT^WT^ and hAAT^CP^ (n = 10 per group, data pooled from three independent experiments).

As shown (Figure 3a), the freshly unstitched wound area size in the vehicle-treated wounds was significantly greater than in both the hAAT^WT^ and hAAT^CP^ groups (Figure 3a *left*). Representative images show partial closure of a vehicle-treated freshly unstitched wound (Figure 3a *right*), and an apparent macroscopic fully closed wound in the hAAT^WT^ and hAAT^CP^ treated groups.

Upon examination of histological samples (Figure 3b), as expected, vehicle-treated wound sections were found to contain a prominent infiltration of leukocytes. The wounds treated with hAAT^CP^ also contained leukocytes, but these appeared to be clustered in compartments that were separated by non-invaded intact tissue. Unexpectedly, the hAAT^CP^ group that displayed advanced wound closure at the macroscopic level depicted an infiltrate of leukocytes which was not dissimilar to the vehicle-treated group; in both, H&E depiction of tissue-invading leukocytes was widespread. 

Relative expression levels of IL-1β and VEGF were determined by RT-PCR (Figure 3c). Expectedly, given that hAAT^WT^ and hAAT^CP^ are essentially anti-inflammatory, both treatments resulted in IL-1β expression that was lower than that observed in the vehicle-treated samples, reaching statistical significance between vehicle-treated and hAAT^CP^-treated wounds. Unexpectedly, in the same samples, VEGF transcript levels were overall elevated compared to the vehicle group.

### 2.4. Inhibitory Effect of Dexamethasone (DEX) Compared to DEX and hAAT^CP^ Treatment

**Figure 4:**
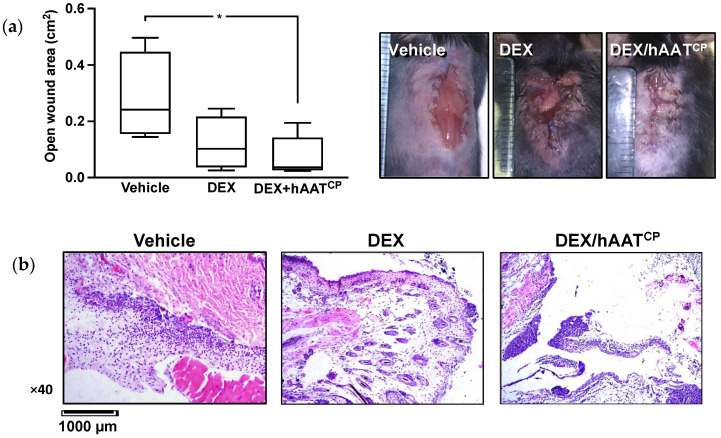
***In vivo wound model: DEX with and without hAAT^CP^*.** Dorsal incisional wounds were inflicted and then immediately stitched closed; 8 h later, stitches were removed and mice were allowed to roam ad libitum for 3 h. Treatments included directly added DEX with or without hAAT^CP^ at the time of wounding. (**a**) Wound area analysis. *Left*, Median; min–max. * *p* < 0.05. *Right*, representative images of wounds in-frame with a physically placed metric ruler. (**b**) Histology. Representative microscope images (optical magnification and scale indicated).

Wound closure in the presence of DEX treatment depicted an expected inferior macroscopic profile compared to the vehicle (Figure 4a). However, in mice that were treated with combined DEX and hAAT^CP^, wound size was reduced 1.58 fold on average (from mean 0.119 to 0.0752 cm^2^, respectively). The mean wound size in vehicle-treated animals was 0.313 cm^2^. Histologically (Figure 4b), DEX-treated wounds exhibited a considerably low leukocyte infiltration, while hAAT^CP^-treated wounds exhibited a prominent infiltrate not dissimilar to that observed in hAAT^CP^ monotherapy in Figure 3b, i.e., widespread leukocyte infiltration in the presence of the non-protease-inhibiting hAAT^CP^.

## 3. Discussion

The present study deepens our understanding of the way hAAT, an acute-phase protein with anti-inflammatory and tissue-protective attributes, navigates through the complex process of wound healing and potentially optimizes its outcomes. Specific attention is given to a less studied feature of this molecule, i.e., its relationship with early incoming neutrophils. Indeed, while both AAT and neutrophils are desired players in the context of tissue repair, an apparent conflict arises: neutrophils require aggressive enzymatic tissue degradation as they infiltrate a tissue, and AAT blocks the tissue-degrading enzymes that neutrophils release [24,25]. It is postulated that the activated neutrophil resolves this conflict by releasing ROS at the spearhead of propagation, which effectively negates the anti-proteolytic activity of AAT [26]. However, the remaining globular surface of AAT is presumably free to bind local molecules, such as those that are abundant at sites of cell injury and that act to exacerbate inflammation [27,28]. Additionally, activated neutrophils express and release their own hAAT [29], and gain a bacterial-burden-reducing capacity by, in part, reduced apoptosis in the presence of hAAT [30,31]. Recent reports propose that neutrophils are pivotal in determining the outcome of wounded tissue; their prolonged presence might impair wound repair [32].

Here, we explored the relationship between neutrophils and AAT in the context of tissue injury by introducing a non-protease-binding mutated form of AAT. The recombinant molecule is positioned intentionally in the violent setting of the first 8 h from skin wounding, a time in which neutrophils predominate, inflammation spreads and tissue destruction outweighs local repair processes [1]. While this specific form of mutated AAT (i.e., recombinant hAAT^CP^) has been identified as anti-inflammatory [26], its impact on early wound repair events has yet to be investigated. Moreover, as both hAAT^WT^ and hAAT^CP^ are effectively anti-inflammatory, outcomes of early wound repair events are hereby contrasted with the effect of dexamethasone on wound repair processes.

Study outcomes show that the epithelial gap and in vivo wound closure rates both display a response to hAAT treatment, which is more pronounced in the early phase of closure, particularly in the case of hAAT^CP^. This corresponds with in vitro findings described by Schuster et al., in which clinical-grade hAAT was added to epithelial cell cultures [33]. However, in the in vivo settings, the question was whether the wounds respond to hAAT by accelerated changes at the cellular level, or rather by a more rapid mechanical constriction. To examine these aspects, wounds were inflicted and then immediately stitched closed, thus canceling the mechanical aspect of wound closure and allowing the focus to shift to underlying changes in cell composition. The rationale for letting the mice roam their cages for three hours after removing the 8-h sutures is that immediately after suture removal, elasticity around the wound renders uniform wound area quantification.

In the epithelial cell gap closure assay, very little difference was found between the impact of hAAT^WT^ and hAAT^CP^ therapies. Although both had enhanced gap closure rates, a slight shift in the timeline featured hAAT^CP^ as leading over hAAT^WT^. This aligns with findings described by Lior et al. [23], in which hAAT^CP^ causes an inflammatory surge to become sharp, short and effective, thus promoting the resolution phase that arrives directly in tandem to the inflammatory phase. At the same time, however, it should be noted that RT-PCR results in the present study show that the expression of IL-1β in wound explants is diminished by both hAAT formulations, while VEGF expression, a classic inflammatory downstream responder, is elevated. This is consistent with accelerated perfusion of skin flaps in hAAT-rich conditions [9] and upregulation of VEGF in angiogenesis studies that examine clinical-grade hAAT [34].

Following on the topic of epithelial gap closure in the presence of hAAT, Schuster et al. show that DEX is unable to interfere with the beneficial outcome of hAAT [33]. Here, the combination of DEX with hAAT^CP^ shows a consistent outcome; that is, wound closure is enabled despite DEX being a clear inhibitor of the process. Nonetheless, it is interesting to observe that the neutrophilic burden remains intact with hAAT^CP^ added to DEX, suggesting that the invasion of neutrophils might not necessarily require inflammation, at least not in the context of DEX. This finding encourages further exploration of dosage and timing combinations between DEX and hAAT so that ideal outcomes are obtained. The capacity of a combination approach to result in superior conditions for wound healing has been suggested in a concentration gradient study: hAAT and DEX were introduced at varying stoichiometric ratios to cultures of macrophages, resulting in a sweet spot of diminished IL-6 and peak IL-1-receptor-antagonist levels [33].

The present study suggests that readily migrating cells (epithelial cells and neutrophils), whether compartmentalized (as in hAAT^WT^-treated animal wounds) or tissue-wide (hAAT^CP^-treated animal wounds) are still favorable over non-migrating cells (DEX-treated cells and animals). It has recently been reported that neutrophils possess at least two phenotypes, of which N2 is anti-inflammatory [35]. It is therefore also possible that neutrophils gain unique phenotypes in the presence of hAAT, a prospect that deserves further study.

In the present study, the inability of local proteases to cleave AATCP already represents a partial mechanism behind the findings. In the publication depicting the profile of hAATCP activity in mice [23] some chemicophysical changes are assumed, as the pharmacokinetics of hAAT^CP^ differ from hAATWT; it is suggested by this that binding to lipid membranes is superior in hAAT^CP^. As the rest of the molecule is presumably intact, we believe that outcomes obtained using hAAT^CP^ highlight the presence of extra-RCL binding sites. A denatured AAT is potentially inflammatory, as inner regions of the molecule might be exposed, and previous attempts to heat-inactivate AAT found that it refolds upon exposure to its native environment, presumably by virtue of its extensive glycosylation branches; it is a very stable molecule. We propose further mutational studies for the exploration of putative binding sites on its surface, as well as experiments using strategic peptidomimetics [27].

Of note, all animals in the study expressed uninterrupted murine AAT (mAAT), and since treatments were topical, it is assumed that they hold negligible effects on liver production of mAAT. Although tempting to employ AAT-KO mice as a categorical control group, we wish to note that such animals exhibit the phenotype of AAT-deficient individuals (excess neutrophilic activity, widespread vasculitis and, depending on the engineered mouse line, aggregate-burdened hepatocytes); we do not assume a surgical cut has an underlying lack of AAT levels, but rather that elevated AAT provides benefit, and further so when it is a form that negates cleavage by target proteases—the only process that literally cuts short the half-life of native AAT. These proteases are common in sites of tissue injury and limit the half-life of local AAT. Unless supplemented by a surge in liver AAT production, local levels are hence limited.

Taken together, the present study offers a possible avenue for driving forward wound recovery, even in the presence of commonly used corticosteroids [36,37,38]. At the molecular level, the finding of more protease-dependent and independent aspects of hAAT joins a long list of dogma-defying studies where the role of protease inhibition in human pathologies might be overemphasized. Finally, the remarkable safety record of repeated infused clinical-grade hAAT for individuals as young as 4 years old [39,40,41], renders the molecule an attractive candidate for promoting wound healing, including via a topical microemulsion preparation.

## 4. Materials and Methods

### 4.1. Animals

Animal studies were approved by the Institutional Animal Care and Use Committee (Approval 48-09-2016) and conducted in line with the *Guide for the Care and Use of Laboratory Animals*, 8th Edition. Six-to-eight-week-old C57BL/6 mice (Envigo+ Laboratories, Inc., Rehovot, Israel) were housed at a vivarium facility.

### 4.2. In Vitro Human Epithelial Gap Repair Assay

In vitro scratch assay was performed using A549 cells (ATCC). Cells were grown to confluence in 24-well plates and uniform wounds were inflicted using a sterile 200 µL pipette tip, thus creating a cell-free area, as described elsewhere [33]. Cultures were washed twice with complete RPMI 1640 supplemented with 2% FCS (both from Biological Industries Inc., Beit Haemek, Israel). Treatments were introduced directly onto cells in 2% FCS; control conditions included media supplemented with no FCS or enriched with 10% FCS. Images were acquired immediately after wounding and then again 3, 6, 12, 24 and 36 h later using a photomicroscope (Zeiss). Images were analyzed by ImageJ, and cell-free areas were marked; outcomes are represented as percent from initial wound area.

### 4.3. In Vivo Wound Models

Mice were anesthetized by isoflurane inhalation (5% to induce, 3% maintenance). Dorsal hair was removed by electric razor and skin was disinfected with chlorhexidine gluconate 0.5% *w*/*v* in 70% *v*/*v* ethanol solution.

#### 4.3.1. Excisional Wound Model

Dorsal skin was gently pulled and raised at the midline to form a twin-layer skinfold. A sterile disposable 5 mm biopsy punch was used to excise opposing skin layers, creating symmetrical full-thickness wounds. Recombinant proteins were directly infiltrated intradermally into wound edges at 100 μL/dose at time of wounding. Wound area was monitored using a digital camera on a fixed stage at indicated time points from wounding. Wound area was marked and determined using ImageJ and Digimizer image analysis software (MedCalc Software, Ostend, Belgium).

#### 4.3.2. Excisional Wound Model with Early Stitch Removal

A 1.5 cm-long dorsal incision was performed, and treatments were introduced onto the wounded surface. Four individual reinforced triangular stitches were immediately applied evenly along the wound using 3-0 sutures. The stitches were removed eight hours later under anesthesia, after which mice were allowed to recover and roam at libitum on a heating pad. Three hours from suture removal, wounds were imaged and wound areas quantified and analyzed. Samples were collected for histology at that time point.

### 4.4. Treatments

Recombinant hAAT formulations were produced and purified as described elsewhere [26]. Dexamethasone (West-Ward Pharmaceuticals, NJ, USA) was administered i.p. at a clinically relevant dose (50 mg/kg [34]). A microemulsion preparation for each recombinant hAAT protein was prepared shortly prior to experiments at 20% oil phase and 80% aqueous phase, as follows. The oil phase contained oleyl alcohol (BASF, NJ, USA), mineral oil, polyoxyl 20 cetostearyl esther (Sigma-Aldrich, Burlington, MA, USA), cetostearyl alcohol (Cognis Deutschland GmbH & Co., KG, Monheim am Rhein, Germany) and sorbitan monostearate (Enco Scientific Services, Petach Tikvah, Israel). Ingredients were heated to 70–75 °C and mixed until full melting. The aqueous phase was a mixture of double-distilled water and preservatives heated to 70–75 °C and mixed until clear. Emulsion: the oil phase was added gradually to the aqueous phase while mixing, followed by 5 min of homogenization and then gradual cooling to 20–25 °C with a gentle magnetic stir. Once at room temperature, recombinant hAAT mixed with double-distilled water was added (0.0001%), and further mixing of the preparation was carried out to achieve homogeneous dispersion of the protein. A vehicle preparation included the above but without added hAAT. All formulations were manufactured shortly prior to the experiments.

### 4.5. Histological Analysis

Animals were sacrificed, and wounds were excised and sliced across the incision line, then immediately immersed in 10% neutral-buffered formalin (Sigma-Aldrich, Rehovot, Israel). Samples were then placed facing the microtome blade for paraffin embedding. Tissues were subsequently cut into 4–6 μm sections, mounted on slides and stained with Hematoxylin and Eosin (H&E; Jackson ImmunoResearch, West Grove, PA, USA). Identification of leukocytes was undertaken as described elsewhere [42].

### 4.6. RT-PCR Gene Expression Analysis

For gene expression analysis of interleukin-1β (IL-1β) and vascular endothelial growth factor (VEGF) in the wounded tissue, RNA extraction from mouse skin was performed using Gynzol^®^ reagent (Invitrogen, Waltham, MA, USA) at 1 mL/well following the manufacturer’s instructions. In brief, tissue was transferred to a Polytron homogenizer, and the homogenized sample was loaded into a 1.5 mL RNase-free microcentrifuge tube. RNA isolation followed manufacturer’s guidelines. RNA was then quantified using a NanoDrop device (Wilmington, DL, USA) and 200 ng of RNA was reverse-transcribed into cDNA using Prime Script RT Reagent kit (Takara, Dalian, China). Quantitative PCR was performed using an RT-PCR system (StepOnePlus™ Real-Time PCR System, Thermo Fisher Scientific corporation, Waltham, MA, USA), and SYBR Premix Ex Taq II (Takara) at a 20 μL volume reaction. CFX96 manager software was used to determine threshold cycle values; 18s rRNA was used as a reference gene. Primers were as follows: murine IL-1β ‘5-CTC CAT GAG CTT TGT ACA AGG-3′ (forward), ‘5-TGC TGA TGT ACC AGT TGG GG-3′ (reverse); murine VEGF, 5′-CTT TAG AGA TCA GCC CAA CC-3′ (forward), 5′-CTA CCC AGA GGG AAG AAA TAA C-3′ (reverse); murine 18s 5′-TCA ACA CAG GGA TCG GAC AAC ACA-3′ (forward), ‘5-GCC TTG GAT CAA GTT CAC AGG CAA-3′ (reverse).

### 4.7. Statistical Analysis

Nonparametric Mann–Whitney or Kaplan–Meier log-rank tests were used to assess statistical significance with 95% confidence interval, as indicated. Results are shown as mean ± SD or mean ± SEM, as indicated. *p* < 0.05 was considered significant. Statistical processing was performed using GraphPad Prism software (GraphPad Software, La Jolla, CA, USA).

## 5. Patents

WO/2018/185756.

## Data Availability

Not applicable.
